# Three-Dimensional Tumor Spheroids Reveal B7-H3 CAR T Cell Infiltration Dynamics and Microenvironment-Induced Functional Reprogramming in Solid Tumors

**DOI:** 10.3390/cells15020169

**Published:** 2026-01-16

**Authors:** Feng Chen, Ke Ning, Yuanyuan Xie, Xiaoyan Yang, Ling Yu, Xinhui Wang

**Affiliations:** 1 Key Laboratory of Luminescence Analysis and Molecular Sensing, Ministry of Education, School of Materials and Energy, Southwest University, Chongqing 400715, China; fchen0@mgh.harvard.edu (F.C.); nk7868@email.swu.edu.cn (K.N.); blsy486900@email.swu.edu.cn (Y.X.); xyang35@mgh.harvard.edu (X.Y.); 2 Division of Gastrointestinal and Oncologic Surgery, Department of Surgery, Massachusetts General Hospital, Harvard Medical School, Boston, MA 02114, USA

**Keywords:** CAR T cells, 3D tumor spheroids, B7-H3, tumor microenvironment, T cell infiltration, immunotherapy

## Abstract

Chimeric antigen receptor (CAR) T cell therapy has demonstrated clinical success in hematologic malignancies but has limited efficacy in solid tumors due to tumor microenvironment (TME) barriers that impede CAR T cell recognition, infiltration, and sustained function. Traditional 2D assays inadequately recapitulate these constraints, necessitating improved in vitro models. This study validated a 3D tumor spheroid platform using an agarose microwell system to generate uniform B7-H3-positive spheroids from multiple solid tumor cell lines, enabling the evaluation of CAR T cell activity. TME-relevant immune modulation under 3D conditions was analyzed by flow cytometry for B7-H3, MHC I/II, and antigen processing machinery (APM), followed by co-culture with B7-H3 CAR T cells to assess cytotoxicity, spheroid integrity, tumor viability, and CAR T cell activation, exhaustion, and cytokine production. Two human cancer-cell-line-derived spheroids, DU 145 (prostate cancer) and SUM159 (breast cancer), retained B7-H3 expression, while MC38 (mouse colon cancer)-derived spheroids served as a B7-H3 negative control. Under 3D culture conditions, DU 145 and SUM159 spheroids acquire TME-like immune evasion characteristics and specifically downregulated MHC-I and APM (TAP1, TAP2, LMP7) with concurrent upregulation of MHC-II and calreticulin. Co-culture showed effective spheroid infiltration, cytotoxicity, and structural disruption, with infiltrating CAR T cells displaying higher CD4^+^ fraction, activation, exhaustion, effector/terminal differentiation, and IFN-γ/TNF-α production. This 3D platform recapitulates critical TME constraints and provides a cost-effective, feasible preclinical tool to assess CAR T therapies beyond conventional 2D assays.

## 1. Introduction

Cancer immunotherapy has revolutionized the treatment landscape of oncology by harnessing the immune system to selectively target malignant cells [1,2,3]. Among the most transformative advances in this field is chimeric antigen receptor T (CAR T) cell therapy, which reprograms autologous T cells to recognize tumor antigens independently of classical antigen presentation via the major histocompatibility complex (MHC) [4,5]. This approach enables potent, antigen-specific cytotoxicity and has shown success in treating hematological malignancies [6,7,8].

In clinical practice, CAR T therapies targeting CD19 have achieved remarkable response rates in patients with relapsed or refractory B cell malignancies, establishing a new paradigm in cancer treatment [9,10,11]. However, translating this success to solid tumors has proven more difficult due to multiple barriers, including tumor heterogeneity, physical exclusion of T cells, and the immunosuppressive tumor microenvironment (TME) [7,12]. A particularly formidable barrier in solid tumors is T cell exhaustion, a dysfunctional state marked by impaired proliferation, reduced cytotoxicity, and sustained expression of inhibitory receptors such as PD-1, TIM-3, and LAG-3 [13,14,15,16]. This exhaustion is often driven by chronic antigen exposure, metabolic stress, and immune evasion mechanisms inherent to the TME [17,18]. Traditional two-dimensional (2D) monolayer cultures fail to replicate these structural and immunological complexities of solid tumor. They lack the three-dimensional (3D) architecture, and diffusion gradients found in solid tumors, limiting their ability to model T cell infiltration and exhaustion accurately. Likewise, animal models, while more representative, are costly, time-consuming, and often unsuited for high-throughput applications [19].

Recent advances in 3D culture systems, particularly tumor spheroids and patient-derived organoids, have provided more physiologically relevant in vitro models for anti-tumor drug screening [20,21,22]. Three-dimensional spheroids better recapitulate key features of the TME, including altered metabolism, chronic hypoxia, nutrient gradients, and compact cellular architecture [19,23]. Typically, 3D tumor spheroids exhibit a central necrotic zone, a middle quiescent zone, and a peripheral proliferative zone [19,24,25,26]. Hypoxic conditions, along with the accumulation of metabolic byproducts, contribute to an acidic and hydrogen peroxide–rich microenvironment [27,28,29]. Moreover, 3D spheroids provide scalable, controllable, and ethically preferable alternatives to animal experiments, reducing variability and ethical concerns while facilitating high-throughput analyses [30,31,32].

Previous studies have explored the use of 3D spheroids to evaluate the cytotoxic effects of CAR T cells against solid tumors [33,34,35,36]. For instance, Chen et al. employed hanging-drop plates to generate BT-474 and SKOV3 spheroids, facilitating assessment of HER2-targeted CAR T cell anti-tumor activity while enabling separation of unbound and dead cells during cytotoxicity assays [36]. Paterson et al. developed a microfluidic immunoassay to evaluate CAR T cell cytotoxicity and targeting specificity using 3D tumor spheroids [37]. Zurowski et al. studied the cytotoxicity of prostate-specific membrane antigen (PSMA) CAR T cell against PC3 prostate cancer spheroids using image cytometry [34]. More recently, Lo et al. utilized magnetic nanoparticles under magnetic field conditions to promote spheroid formation from non-small cell lung cancer (NSCLC) patient-derived xenografts for studying T cell infiltration [38]. Those investigations have predominantly focused on CAR T cell killing efficacy with limited exploration of tumor-infiltrating CAR T cell phenotypes, particularly their exhaustion status and functional characteristics following co-culture with 3D spheroids. In addition, it is important to consider the immunological context that influences CAR T cell performance in solid tumors. While CAR T cells recognize cell surface antigens independently of classical MHC-peptide presentation, tumor MHC expression can indirectly influence CAR T cell performance by modulating local inflammatory responses and the activity of CD4^+^/CD8^+^ T cells. Loss of MHC-I/II expression represents a common immune escape mechanism associated with non-inflamed (“cold”) tumors that exhibit reduced cytokine production and increased immunosuppressive cell infiltration. The capacity of 3D spheroids to recapitulate key solid tumor TME features, including hypoxia and acidosis, provides a compelling rationale for investigating how these conditions influence tumor antigen expression levels, immune-related molecules (MHC-I and MHC-II), and antigen presentation machinery (APM) components in 3D tumor spheroids. Such models enable detailed examination of how TME characteristics and immune status impact CAR T cell infiltration, cytotoxicity, and functional dysfunction under physiologically relevant conditions.

Building on these advantages, we employed an agarose microwell-based spheroid culture platform to generate uniform tumor spheroids and facilitate efficient CAR T cell co-culture. In this study, B7-H3-expressing 3D tumor spheroids were established and systematically characterized, followed by comprehensive assessment of B7-H3-targeted CAR T cell interactions with these spheroids. Parameters evaluated included tumor antigen B7-H3 expression, MHC-I and MHC-II levels, APM components (TAP1, TAP2, LMP7), T cell infiltration patterns, and phenotypic characteristics of tumor-infiltrating CAR T cells during 3D co-culture. The cytotoxicity of B7-H3 CAR T cells on 3D tumor spheroids were quantified through analysis of spheroid structural integrity and live/dead cell staining. Additionally, both tumor-infiltrating and non-engaged (free) CAR T cells were isolated from the co-culture system to evaluate exhaustion-related markers, enabling investigation of how chronic antigen exposure, hypoxia, and acidosis within the 3D tumor spheroid microenvironment contribute to CAR T cell exhaustion.

## 2. Materials and Methods

### 2.1. Cell Lines and Reagents

*Cell lines and culture condition*: Human prostate cancer cell line DU 145 was obtained from the American Type Culture Collection (ATCC), human triple-negative breast cancer SUM159 was obtained from Asterand Bioscience (Detroit, MI, USA), and murine colon adenocarcinoma cell line MC38 was from MilliporeSigma (Burlington, MA, USA). The information related to those cells can be found at Research Resource Identifier (RRID) database [39]. The RRID of those cells are: DU 145: CVCL_0105 SUM 159: CVCL_5423 MC38: CVCL_B288. DU 145-mCherry cells, previously established in our laboratory, were also utilized. All cells were cultured in RPMI-1640 (Corning, Manassas, VA, USA) supplemented with 10% fetal bovine serum (FBS, Biowest, Riverside, MO, USA).

*General reagents*: Click’s medium and phosphate-buffered saline (PBS) were purchased from Sigma-Aldrich (St. Louis, MO, USA). Molecular biology grade agarose was sourced from MedChemExpress (Monmouth Junction, NJ, USA). Collagenase type IV was obtained from Gibco (Thermo Fisher Scientific, Waltham, MA, USA). PKH 67 (green) fluorescent cell linker was ordered from Sigma-Aldrich (cat# PKH67GL-1KT, St. Louis, MO, USA). DAPI (4′,6-diamidino-2-phenylindole, 1 mg/mL) was purchased from Invitrogen (Thermo Fisher Scientific, Waltham, MA, USA). Cyto-Fast™ Fix/Perm Buffer Set (cat# 426803) was obtained from BioLegend (San Diego, CA, USA).

### 2.2. Generation and Culture of B7-H3 CAR T

B7-H3 CAR T cells expressing the single-chain variable fragment (scFv) derived from the B7-H3-specific 376.96 monoclonal antibody [40] were generated using a standardized protocol. Peripheral blood mononuclear cells (PBMCs) were obtained from healthy donors (Research Blood Components, Watertown, MA, USA).

*Activation and transduction protocol*: On day 0, PBMCs (1 × 10^6^ cells/well) were activated in 24-well non-treated plates (#351147, Corning) that had been pre-coated with anti-human CD3 (cat# 130-093-387, clone: OKT3, 1 µg/mL, Miltenyi Biotec, Waltham, MA, USA) and anti-human CD28 antibodies (cat# 556620, clone: CD28.2, 1 µg/mL, BD Biosciences, San Diego, CA, USA), using complete medium comprising 45% RPMI1640, 45% Click’s medium, 10% FBS, 1% penicillin, and 1% streptomycin (Corning). Activated T cells were expanded with IL-7 (10 ng/mL) and IL-15 (5 ng/mL, both PeproTech, Cranbury, NJ, USA) supplementation, designated as CAR T medium. On day 3, activated T cells were transferred to RetroNectin-coated 24-well plates (Takara Bio Inc., Shiga, Japan) containing retroviral particles encoding the CAR construct for transduction.

*Expansion and maintenance*: On day 4, transduced T cells were collected and reseeded into tissue culture-treated 24-well plates (#353047, Corning) at 5 × 10^5^ cells/well in 0.5 mL activated T-cell suspension supplemented with 1.5 mL fresh CAR T medium. Transduction efficiency was evaluated on day 6 using FITC-labeled human B7-H3 (4Ig)/B7-H3b protein (0.5 µg/100 µL, cat# B7B-HF2E7, ACRO Biosystem, Beijing, China). Subsequently, CAR T cells were maintained at 1 × 10^6^ cells/well in 2 mL fresh CAR T medium with medium exchanges every three days until collection, aliquoting, and cryopreservation in liquid nitrogen for subsequent experiments.

### 2.3. Generation and Characterization of 3D Tumor Spheroids in Agarose Micro-Wells

**Scheme 1:**
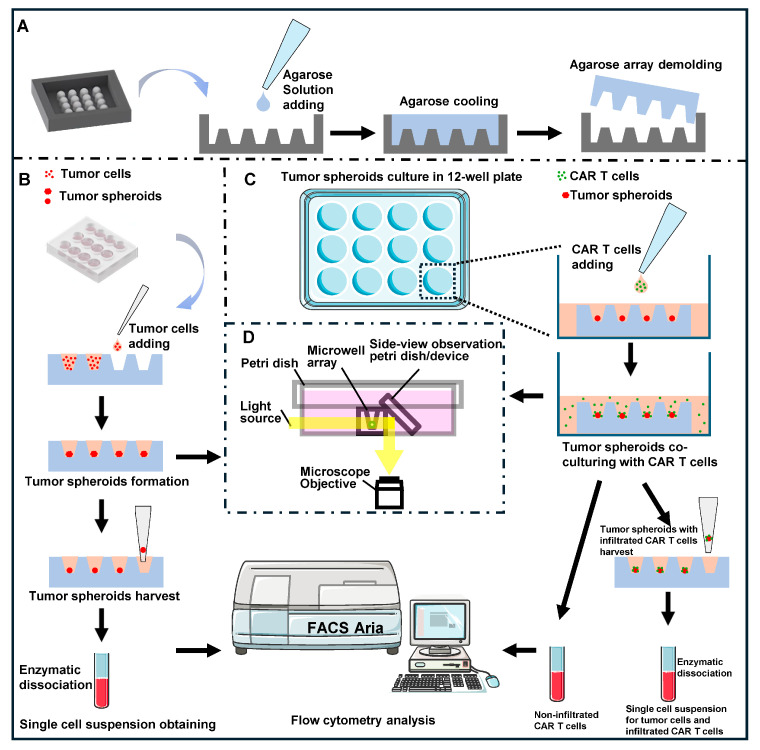
**Overview of the agarose-array-based spheroid culture and CAR T-cell infiltration assay.** (**A**) Fabrication of agarose microwell array from 3D-printed mode. (**B**) In situ formation of tumor spheroids within the agarose array and subsequent harvesting for FACS analysis. (**C**) Co-culture of CAR T cells with tumor spheroids in the agarose microwell array and harvest for further FACS analysis. (**D**) In situ side-view imaging device for real-time observation of 3D spheroids.

*Agarose micro-well array fabrication*: Agarose micro-well arrays were generated following a previously reported protocol [41]. The fabrication mold containing U-shaped columns (2 mm height, 2 mm diameter) was created using a Digital Light Processing 3D printer (SprintRay Inc., Los Angeles, CA, USA). As shown in Scheme 1A, 1 mL of 2% agarose solution was added to cover the 3D printed mold and allowed to solidify at room temperature (25 °C). The agarose base was then carefully separated from the mold to obtain the micro-well array.

*Spheroid culture*: Agarose micro-well arrays were placed inside 12-well cell culture plates, and 8 μL of cell suspension (1 × 10^4^ cells/well) was added to each micro-well. Subsequently, 600 μL of culture medium was added to each well of the 12-well plate. Cells were cultured at 37 °C and 5% CO_2_, with medium changes every 2 days. Spheroid morphology was monitored using bottom-view and side-view imaging with a home-made in situ side-view imaging device [25] under microscope (Eclipse TS100, Tokyo, Nikon, Japan). Spheroids were harvested after 1, 3, 5, and 7 days of culture for comprehensive characterization (Scheme 1B), including viability assessment, B7-H3 expression, and APM analysis.

*Viability assessment* via *live/dead staining*: Spheroids were rinsed twice with PBS and incubated with Calcein-AM/propidium iodide (PI) Live/Dead Cell Staining Kit (Beyotime Biotechnology, Shanghai, China cat#C2015S, 1:1000 dilution) for 60 min at 37 °C with 5% CO_2_. Following PBS washes, spheroids were imaged using fluorescence microscopy (TS100-F, Nikon, Japan).

*Viability assessment* via *flow cytometry analysis*: Spheroids were dissociated into single-cell suspensions using Collagenase IV (100 U/mL in HBSS, Cat#17104-019, Gibco, Waltham, MA, USA), washed with FACS buffer (0.5% BSA in PBS buffer), and stained with DAPI before acquisition. DAPI^−^ cells were gated as viable cells.

*Surface marker analysis:* For B7-H3, MHC-I, and MHC-II expression profiling, spheroids were harvested, washed with cold PBS, and dissociated into single cells using Collagenase IV (100 U/mL in HBSS). Single-cell suspensions were incubated with in-house monoclonal antibodies (mAbs) include mouse anti-B7-H3 mAb 376.96 with isotype control mAb F3C25 for B7-H3 detection [42], or MHC class I-specific mAb TP25.99.8.4, MHC class II-specific mAb LGII-612.14 with isotype-matched IgG1 mAb (MK2-23) serving as specificity control [43] for 1 h at 4 °C. After FACS buffer washes, cells were incubated with R-Phycoerythrin-conjugated secondary antibody (R-Phycoerythrin AffiniPure F(ab’)2 fragment goat anti-mouse IgG (H + L), Jackson ImmunoResearch, West Grove, PA, USA, cat# 115-116-146) for 30 min at 4 °C, washed, and analyzed by flow cytometry. Murine MHC-I was stained with Brilliant Violet 421 anti-mouse H-2Kb (BioLegend, San Diego, CA, USA, cat# 116525, clone: AF6-88.5).

*Intracellular staining*: Single-cell suspensions were prepared as above, washed with 1% BSA/PBS buffer, and fixed with 2% formaldehyde-paraformaldehyde for 20 min at room temperature. After washing, cells were resuspended in 0.5% BSA/PBS buffer, denatured with microwave treatment (200 W, 50 s), and immediately placed on ice for 10 min. Cells were then permeabilized with 0.1% saponin/1% BSA/PBS buffer for 30 min at room temperature and incubated with intracellular APM antibodies, including calreticulin-specific mAb TO-11, TAP1-specific mAb NOB1, TAP2-specific mAb NOB2, and LMP7-specific mAb HB2, MHC class I-specific mAb TP25.99.8.4, MHC class II-specific mAb LGII-612.14 with isotype-matched IgG1 mAb (MK2-23) serving as specificity control [43] for 30 min at room temperature. Following saponin buffer washes, cells were stained with Allophycocyanin (APC) AffiniPure® F(ab’)_2_ Fragment Goat Anti-Mouse IgG (H+L) (Jackson ImmunoResearch, West Grove, PA, USA, cat# 115-136-146) secondary antibody, washed sequentially with saponin buffer and FACS buffer, and analyzed by flow cytometry.

### 2.4. Co-Culture of CAR T Cells with 2D Cultured Tumor Cells

Tumor cells were seeded at 1 × 10^4^ cells per well in 96-well plates and incubated overnight. B7-H3 CAR T cells were added at effector-to-target (E:T) ratios of 4:1, 2:1, 1:1, 1:2, 1:4, and 1:8 in complete CAR T medium without cytokines. After 24 h co-culture, cell morphology was imaged at 200× magnification to assess CAR T-mediated killing. Cytotoxicity was quantified using MTT assay: wells were washed with PBS, incubated with MTT solution (0.5 mg/mL, Sigma cat#M6494) for 4 h in darkness, and formazan crystals were solubilized in DMSO. Absorbance was measured at 570 nm using an Epoch microplate reader (BioTek instrument, Winooski, VT, USA). Target cell viability (%) was calculated as: (OD_CAR T+targets_ − OD_CAR T only_)/OD_targets only_ × 100%. Triplicate assays were performed.

### 2.5. Co-Culture of CAR T Cells with 3D Tumor Spheroids

*Co-culture setup and morphological assessment*: As illustrated in Scheme 1C, B7-H3 CAR T cells were added to agarose microwells containing 3D tumor spheroids at defined E:T ratios (4:1, 2:1, 1:1) and maintained in CAR T complete medium. Morphological changes in spheroid lateral dimensions and thickness under CAR T treatment were monitored using the side-view microdevice (Scheme 1D) [25].

*Cytotoxicity quantification*: CAR T killing effects on spheroids were quantified by flow cytometry. Spheroids were dissociated into single-cell suspensions using Collagenase IV (100 U/mL in HBSS), stained with APC anti-human CD45, and treated with DAPI (0.5 µL of 1 mg/mL) before acquisition. Live tumor cells were identified as CD45^−^DAPI^−^ after debris exclusion and singlet gating.

*CAR T cell infiltration assessment*: B7-H3 CAR T cells were labeled with PKH67 Green Fluorescent Cell Linker Kit (Phanos Technologies, Inc., Sanit Louis, MO, USA) following manufacturer’s protocol: 2 × 10^7^ cells were washed with serum-free RPMI 1640, resuspended in 1 mL Diluent C, and mixed with 1 mL PKH67 dye solution (2 µM final concentration). After 3 min incubation at room temperature, the reaction was quenched with FBS, and cells were washed three times with complete medium. Labeled CAR T cells were co-cultured with 5-day-old DU 145-mCherry spheroids to assess infiltration patterns.

### 2.6. Phenotypic Analysis of Infiltrated Versus Non-Infiltrated CAR T Cells

*Cell harvest and separation*: As illustrated in Scheme 1C, B7-H3 CAR T cells were added to agarose microwells containing 3D tumor spheroids and co-cultured in CAR T complete medium for 3 days. Then, non-infiltrated CAR T cells were collected from culture medium and PBS washes of spheroids. Infiltrated CAR T cells were obtained by dissociating spheroids with Collagenase IV to generate single-cell suspensions containing both tumor and CAR T cells.

*Flow cytometry staining and analysis*: Harvested cells were washed with FACS buffer and stained at 4 °C for 30 min with marker panels (Table 1). For intracellular cytokine detection, cells were fixed and permeabilized using Fix/Perm buffer (4 °C, 30 min), then stained with TNF-α-PE and IFN-γ-APC (room temperature, 30 min). After washing with Perm/Wash buffer and FACS buffer, cells were resuspended in 300 µL FACS buffer and acquired on a BD FACSAria™ flow cytometer (BD Biosciences, San Jose, CA, USA). Commercial antibodies used above are all from BioLegend (San Diego, CA, USA, 1:100 dilution).

*Data analysis*: Flow cytometry data were analyzed using FlowJo software (v10.8.1, BD Biosciences, San Jose, CA, USA). Sequential gating included: FSC/SSC for debris exclusion, singlet discrimination (FSC-H vs. FSC-A), dead cell exclusion (DAPI^−^), and CAR T cell identification (CD45^+^CD3^+^). CAR T cells were subdivided into CD4^+^ and CD8^+^ subsets and analyzed for activation markers (CD69, CD25), memory phenotype (CD45RA, CD62L), exhaustion markers (PD-1, LAG-3), and intracellular cytokines (TNF-α, IFN-γ). Non-co-cultured, non-infiltrated, and infiltrated CAR T cell populations were processed and analyzed in parallel.

### 2.7. Statistic Analysis

All statistical analyses were performed using GraphPad Prism software (version 10.4.0, GraphPad Software). Data are presented as mean ± standard deviation (SD) unless otherwise stated. For comparisons involving more than two groups, one-way or two-way analysis of variance (ANOVA) followed by Tukey’s or Sidak’s post hoc tests was applied as appropriate. *p* < 0.05 was considered statistically significant. The number of biological replicates (*n*) and the statistical tests used are indicated in the figure legends.

## 3. Results and Discussion

### 3.1. Three-Dimensional Tumor Spheroids Demonstrate Enhanced B7-H3 Expression

**Figure 1:**
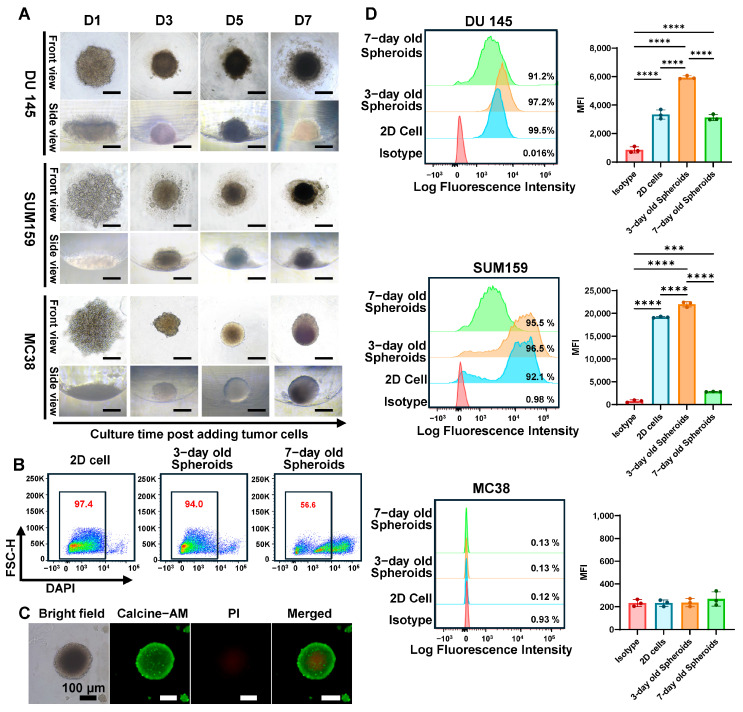
**Agarose microwell supported 3D tumor spheroids growth.** (**A**) Time-laps morphological changes and structural evolution during spheroids formation, scale bar 200 µm; (**B**) DU 145 spheroids’ viability quantified by flow cytometer counted DAPI negative cell population; (**C**) DU 145 spheroid’s viability characterized by Calcein-AM (green) and PI (red) fluorescent staining, scale bar 100 µm; (**D**) Characterization of B7-H3 expression on 3D spheroids and 2D cultured DU 145 cells, SUM159 cells, and MC38 cells by flow cytometry. One-way ANOVA followed by Tukey’s multiple comparisons test was applied, *n* = 3. * indicates *p* < 0.05, ** indicates *p* < 0.01, *** indicates *p* < 0.001, **** indicates *p* < 0.0001.

To establish a physiologically relevant in vitro model for evaluating CAR T cell function against solid tumors, we generated 3D tumor spheroids from multiple solid tumor cell lines (DU 145, SUM159, MC38) using an agarose microwell platform. Time-lapse imaging revealed distinct phases of spheroid assembly and maturation (Figure 1A). Upon seeding in non-adhesive agarose microwells, cells initially formed gravity-driven aggregates at the well center, creating disk-like structures with lateral dimensions of approximately 450 μm but minimal thickness (~50 μm) at day 1. This initial aggregation phase was followed by active cellular reorganization and compaction, progressively increasing aggregate thickness to 200 μm while reducing lateral diameter. By day 3, spheroids achieved a morphology with lateral diameter of 300 μm and thickness of 320 μm, yielding a length-to-height (L/H) ratio approaching 1.0, characteristic of spheroidal structures. Both bottom- and side-view microscopy confirmed dense cellular architecture and spherical morphology.

Next, viability and spatial organization were analyzed. Flow cytometric analysis of DAPI-stained dissociated spheroids demonstrated a time-dependent decrease in viable cell proportion from 97.4% (day 1) to 56.6% (day 7) in DU 145 spheroids (Figure 1B). Complementary live/dead staining using Calcein-AM (viable cells, green) and PI (dead cells, red) revealed spatially distinct zones, with PI-positive cells predominantly localized to the spheroid core (Figure 1C). This spatial organization recapitulates the characteristic architecture of solid tumors, featuring a central necrotic/hypoxic core, intermediate quiescent zone, and peripheral proliferative zone. These gradients arise from limited diffusion of nutrients and oxygen into the spheroid interior.

Given the critical importance of target antigen expression for CAR T cell recognition and cytotoxicity, we systematically evaluated B7-H3 expression patterns in 2D versus 3D culture conditions. Initial screening confirmed that DU 145 and SUM159 cell lines exhibited high baseline B7-H3 expression in 2D culture, while MC38 (mouse colon cancer) cells lacked detectable B7-H3 expression and served as a negative control (Figure 1D). Notably, 3D spheroid culture significantly modulated B7-H3 expression dynamics compared to 2D conditions. DU 145 spheroids maintained consistently high B7-H3 positivity (>90%) throughout the culture period: 99.5% (day 0), 97.2% (day 3), and 91.2% (day 7). More importantly, mean fluorescence intensity (MFI) analysis revealed enhanced B7-H3 expression in early spheroids, peaking at day 3 (MFI = 5937 ± 118.2) compared to 2D culture levels (MFI =3338.67 ± 325.09) (Figure 1D). By day 7, both B7-H3 positivity and MFI decreased (MFI = 3124 ± 214.6), likely attributable to the expanding necrotic core. Similar patterns were observed in SUM159 spheroids, which maintained high B7-H3 expression in early 3D culture (95.5% at day 3) before declining substantially by day 7, correlating with increased cell death and spheroid maturation (Figure 1D). Conversely, MC38 cells showed negligible B7-H3 expression across all conditions (Figure 1D), confirming their utility as negative controls for subsequent CAR T cell specificity studies. These findings demonstrate that 3D spheroid culture not only preserves but can enhance target antigen expression while simultaneously creating physiologically relevant microenvironmental gradients.

### 3.2. Three-Dimensional Spheroid Culture Induces MHC-I Downregulation and Antigen Processing Machinery Defects While Upregulating MHC-II

Building on our observation that 3D spheroids recapitulate solid tumor architecture and enhance B7-H3 expression, we next investigated how the complex microenvironmental conditions within spheroids influence immune recognition pathways. This analysis is particularly critical for understanding CAR T cell function, as while CAR T cells recognize antigens independently of MHC presentation, the broader immune context shaped by MHC expression and antigen processing machinery can significantly impact local inflammatory responses and overall therapeutic efficacy.

**Figure 2:**
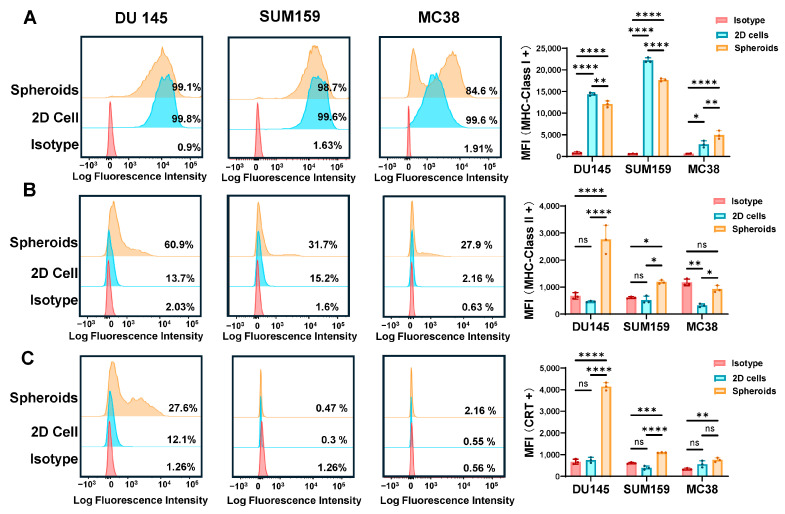
**MHC class-I, II and CRT modulation in 3D spheroids (3 days old).** Expression of (**A**) MHC-I, (**B**) MHC-II, and (**C**) calreticulin (CRT) in 3D spheroids quantified by frequency and MFI. *n* = 3; Comparisons in each tumor cell line were performed using one-way ANOVA followed by Sidak’s multiple comparisons test. * indicates *p* < 0.05, ** indicates *p* < 0.01, *** indicates *p* < 0.001, **** indicates *p* < 0.0001, ns = not significant.

First, MHC class I and II expression patterns in 3D culture was studied. Flow cytometric analysis revealed striking alterations in MHC expression patterns between 2D and 3D culture conditions across all tested cell lines. Most notably, all three spheroid models exhibited significantly impaired MHC-I expression compared to their 2D counterparts (Figure 2A), suggesting compromised classical antigen presentation capabilities during spheroid cultivation. While DU 145 and SUM159 spheroids maintained high MHC-I positivity (>99%), both showed substantial reductions in mean fluorescence intensity, indicating decreased per-cell MHC-I expression levels. MC38 spheroids demonstrated more pronounced impairment, with flow cytometry revealed bimodal populations: a smaller fraction of cells that had significantly reduced MHC-I expression, and a larger fraction that retained higher MFI levels. Overall, MHC-I-positive cell proportions in MC38 spheroids declined from 99.6% to 84.6%. These findings suggest that 3D culture conditions differentially affect MHC-I expression across cancer cell lines, with MC38 spheroids showing the most drastic alterations. To determine whether heterogeneous MHC expression patterns exist among cells at different spatial locations within 3D spheroids, future studies should employ immunohistochemical staining to evaluate MHC-I and MHC-II expressions in cells isolated from different regions of the spheroids. This approach would elucidate the impact of spatial gradients in oxygen/nutrient deprivation, acidic microenvironment, and other local factors on MHC expression.

Conversely, MHC-II expression exhibited an upregulation in spheroids, most prominently in DU 145 cultures (Figure 2B). The proportion of MHC-II-positive DU 145 cells increased dramatically from 13.7% in 2D culture to 60.9% in 3D spheroids, accompanied by significantly elevated MFI values. This unexpected MHC-II upregulation may represent a compensatory immune activation response to microenvironmental stress or reflect altered cellular differentiation states under 3D culture conditions. Calreticulin (CRT) analysis provided additional insights into spheroid-associated immune signaling alterations. CRT expression was markedly induced in DU 145 spheroids (27.6%) compared to 2D cultures (12.1%), indicating the development of immunogenic cell stress responses characteristic of solid tumor microenvironments (Figure 2C).

To elucidate the mechanistic basis for observed MHC-I downregulation, we systematically analyzed key APM components essential for MHC-I-restricted antigen presentation: TAP1, TAP2, and the immunoproteasome subunit LMP7. These molecules are critical for peptide transport from the cytosol to the endoplasmic reticulum and for generating MHC-I-binding peptides, respectively.

**Figure 3:**
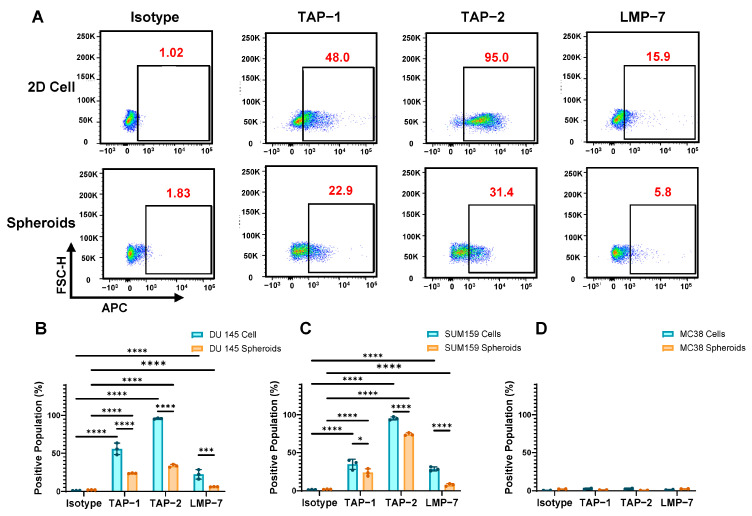
**APM downregulation in 3D spheroids.** (**A**) Representative flowcytometry histograms of DU 145 spheroids. Quantification of APM (TAP1, TAP2, and LMP-7) expression in (**B**) DU 145, (**C**) SUM 159, and (**D**) MC38 spheroids. *n* = 3, two-way ANOVA was performed followed by Tukey’s multiple comparisons test, * indicates *p* < 0.05, *** indicates *p* < 0.001, **** indicates *p* < 0.0001.

DU 145 2D cultures exhibited robust baseline expression of all APM components: TAP1 (48%), TAP2 (95%), and LMP7 (15.9%) (Figure 3A). However, 3D spheroid culture induced dramatic reductions across all components: TAP1 (22.9%), TAP2 (31.4%), and LMP7 (5.8%). Quantitative analysis of day-3 spheroids confirmed statistically significant decreases in all APM components compared to 2D controls: TAP1 (23.3 ± 0.4% vs. 55.6 ± 7.6%), TAP2 (33.2 ± 1.9% vs. 95.8 ± 0.8%), and LMP7 (6.0 ± 0.2% vs. 22.0 ± 6.1%) (all *p* < 0.05; Figure 3B). SUM159 spheroids exhibited analogous APM reductions relative to 2D cultures (Figure 3C), indicating that this phenomenon might represent a general response to 3D culture conditions. MC38 cultures displayed negligible APM expression in both 2D and 3D conditions (Figure 3D).

These comprehensive findings reveal that 3D spheroid culture induces a complex reprogramming of immune recognition machinery. The coordinated downregulation of MHC-I and APM components, coupled with MHC-II upregulation and CRT induction, suggests that spheroid-cultured tumor cells develop a distinct immunophenotype characterized by: (1) impaired classical CD8^+^ T cell recognition due to defective MHC-I presentation, (2) enhanced potential for CD4^+^ T cell activation through increased MHC-II expression. This immunophenotypic transformation likely results from the combined effects of hypoxia, nutrient limitation, acidosis, and metabolic stress within the 3D architecture—conditions that collectively create a “cold” tumor microenvironment with reduced inflammatory signaling. Such conditions are highly relevant for CAR T cell therapy evaluation, as they represent the challenging microenvironmental landscape that CAR T cells must navigate in solid tumors.

Based on these characterization results, we established an optimized experimental framework using day-3 spheroids, which demonstrated peak B7-H3 expression while exhibiting mature microenvironmental characteristics. DU 145 and SUM159 spheroids serve as B7-H3-positive models with distinct immune profiles, while MC38 spheroids provide essential B7-H3-negative controls for assessing CAR T cell specificity.

### 3.3. B7-H3 CAR T Cells Demonstrate Antigen-Specific Cytotoxicity and Progressive Infiltration-Mediated Disruption of Tumor Spheroid Architecture

Having established that 3D spheroids recapitulate key solid tumor characteristics including enhanced B7-H3 expression and compromised immune recognition machinery, we next evaluated the functional capacity of B7-H3 CAR T cells to recognize, infiltrate, and eliminate tumor cells within this physiologically relevant microenvironment.

**Figure 4:**
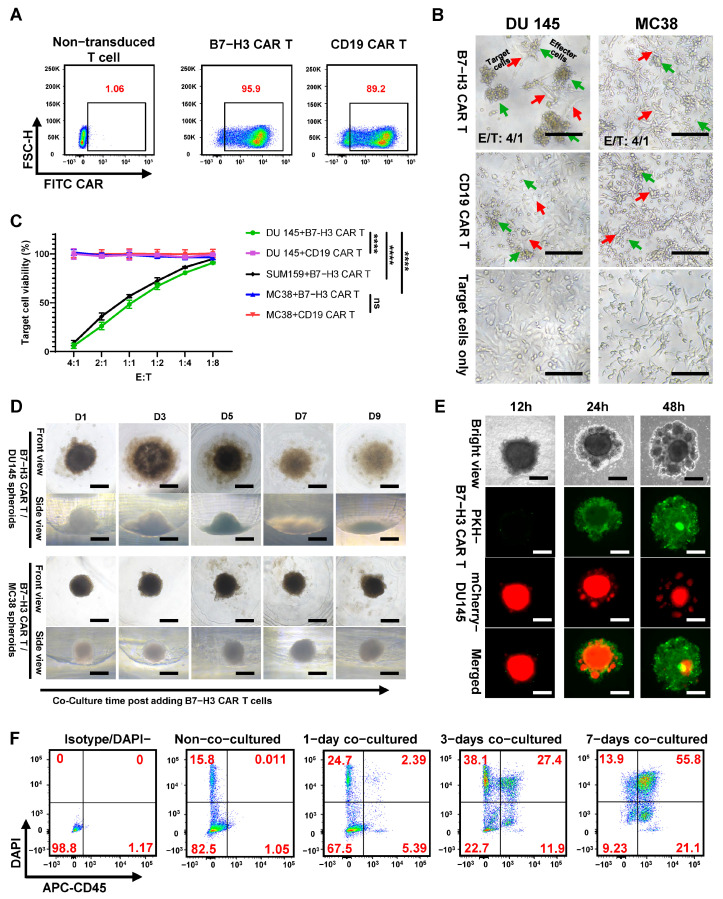
**B7-H3 CAR T cells demonstrate antigen-specific cytotoxicity and progressive infiltration-mediated disruption of tumor spheroid architecture**. (**A**) Flow cytometry confirming CAR expression in transduced T cells; (**B**) Phase-contrast images showing B7-H3 CAR T–mediated killing of DU 145 (B7-H3^+^) but not MC38 (B7-H3^−^) cells at E:T = 4:1 in 2D assays. Red arrows denote target cells (tumor cells) and green arrows denote effector cells (CAR T cells); (**C**) MTT assay demonstrating dose-dependent killing of DU 145 and SUM159 by B7-H3 CAR T cells in 2D assays. Two-way ANOVA followed by Sidak’s post hoc tests was applied as appropriate. **** indicates *p* < 0.0001; (**D**) Bright-field images showing structural changes in DU 145 and MC38 spheroids co-cultured with B7-H3 CAR T cells over 9 days; (**E**) Fluorescence images of DU 145-mCherry spheroids (red) co-cultured with PKH67-labeled B7-H3 CAR T cells (green) for 12 h, 24 h, and 48 h; (**F**) Flow cytometry analysis of B7-H3 CAR T-mediated killing of DU 145 spheroids. Scale bar, 200 µm, ns = not significant.

B7-H3 CAR T cells were successfully generated with high transduction efficiency, as confirmed by flow cytometric analysis using FITC-labeled B7-H3 protein. This approach directly validates functional CAR expression by demonstrating specific antigen recognition capability. Flow cytometry revealed that 95.9% of transduced T cells expressed the B7-H3-targeting CAR, while the control CD19 CAR T cells showed 89.2% transduction efficiency using FITC-labeled CD19 protein (Figure 4A). These high transduction rates ensured robust CAR T cell populations for subsequent functional analyses.

To validate antigen specific cytotoxicity, standard 2D co-culture assays were conducted using the characterized cell lines: B7-H3-positive DU 145 and SUM159 cells, and B7-H3-negative MC38 cells. Time-lapse imaging revealed dramatic morphological evidence of specific cytotoxicity, with B7-H3 CAR T cells (green arrows) effectively eliminating DU 145 target cells (red arrows) within 24 h at an E:T ratio of 4:1 (Figure 4B). Quantitative MTT viability assays confirmed selective tumor cell elimination: B7-H3 CAR T cells demonstrated dose-dependent cytotoxicity against both DU 145 and SUM159 cells while leaving MC38 cells unaffected (Figure 4C). Importantly, CD19 CAR T cells showed no cytotoxic activity against any tested cell line, confirming the antigen-specific nature of the observed killing and validating our experimental controls.

The transition from 2D to 3D evaluation revealed dramatically different CAR T cell-tumor interaction dynamics. Time-lapse bottom-view imaging of DU 145 spheroids co-cultured with B7-H3 CAR T cells demonstrated progressive loss of compact, spherical architecture over 9 days (Figure 4D). Spheroids exhibited increasing horizontal diameter expansion from 300 μm to 450 μm, accompanied by the appearance of dispersed cells surrounding the original spheroid boundary, indicating structural disintegration rather than simple volume reduction. Side-view imaging provided even more dramatic evidence of architectural collapse, revealing the 3D nature of spheroid destruction that cannot be appreciated in conventional 2D assays (Figure 4D). This progressive disintegration likely results from the combined effects of direct cytotoxicity and mechanical disruption caused by CAR T cell infiltration and activation within the confined spheroid space. The prolonged 9-day observation period, substantially longer than typical 2D assays, provides a clinically relevant timeframe for studying CAR T cell persistence and potential exhaustion under chronic antigen exposure conditions. MC38 spheroids lacking B7-H3 expression remained structurally intact throughout the 9-day co-culture period, confirming that observed effects were antigen-dependent (Figure 4D).

To understand the mechanistic basis of spheroid disruption, we employed dual-fluorescence tracking using PKH67-labeled B7-H3 CAR T cells (green) and DU 145-mCherry tumor spheroids (red). At 12 h post-co-culture, CAR T cells predominantly accumulated at the spheroid periphery with limited penetration into the compact tumor mass, suggesting initial recognition and adhesion phases. This peripheral accumulation likely reflects the time required for CAR T cell activation and the physical barriers presented by the dense spheroid architecture. By 24 h, CAR T cells demonstrated progressive infiltration throughout the outer spheroid layers, establishing intimate contact with tumor cells and coinciding with early morphological disruption. Most remarkably, at 48 h, CAR T cells had achieved deep penetration into the spheroid core, accompanied by marked structural disintegration and pronounced reduction in mCherry signal intensity, indicating extensive tumor cell elimination (Figure 4E).

Lastly, flow cytometric analysis of dissociated spheroids provided quantitative validation of the morphological observations. Using CD45 and DAPI staining to distinguish CAR T cells from viable tumor cells, we tracked cytotoxicity kinetics at an E:T ratio of 4:1. Control DU 145 spheroids maintained 82.5% viable tumor cells (CD45^−^DAPI^−^), while B7-H3 CAR T cell co-culture resulted in progressive tumor cell elimination: 67.5% viable cells at day 1, 22.7% at day 3, and only 9.23% at day 5 (Figure 4F). The CAR T infiltration pattern and time-dependent cytotoxicity demonstrate sustained CAR T cell activity within the challenging 3D microenvironment, despite the presence of hypoxic zones, nutrient gradients, and compromised antigen presentation machinery identified in our previous characterization.

### 3.4. Tumor Microenvironment-Induced Phenotypic and Functional Reprogramming of Infiltrated B7-H3 CAR T Cells

Having demonstrated B7-H3 CAR T cell infiltration and cytotoxic activity within 3D tumor spheroids, we next investigated how the complex microenvironmental conditions, including altered MHC expression, compromised APM, and metabolic stress, influence CAR T cell phenotype and function. This spatially resolved analysis comparing tumor-infiltrating versus non-infiltrating CAR T cells provides critical insights into the dynamic adaptations that occur when CAR T cells encounter physiologically relevant solid tumor conditions.

To dissect location-specific CAR T cell responses, we employed a three-way comparison strategy: (1) non-co-cultured CAR T cells serving as baseline controls, (2) free-floating (non-infiltrating) CAR T cells collected from culture supernatants, and (3) tumor-infiltrating CAR T cells isolated from dissociated spheroids after co-culture.

**Figure 5:**
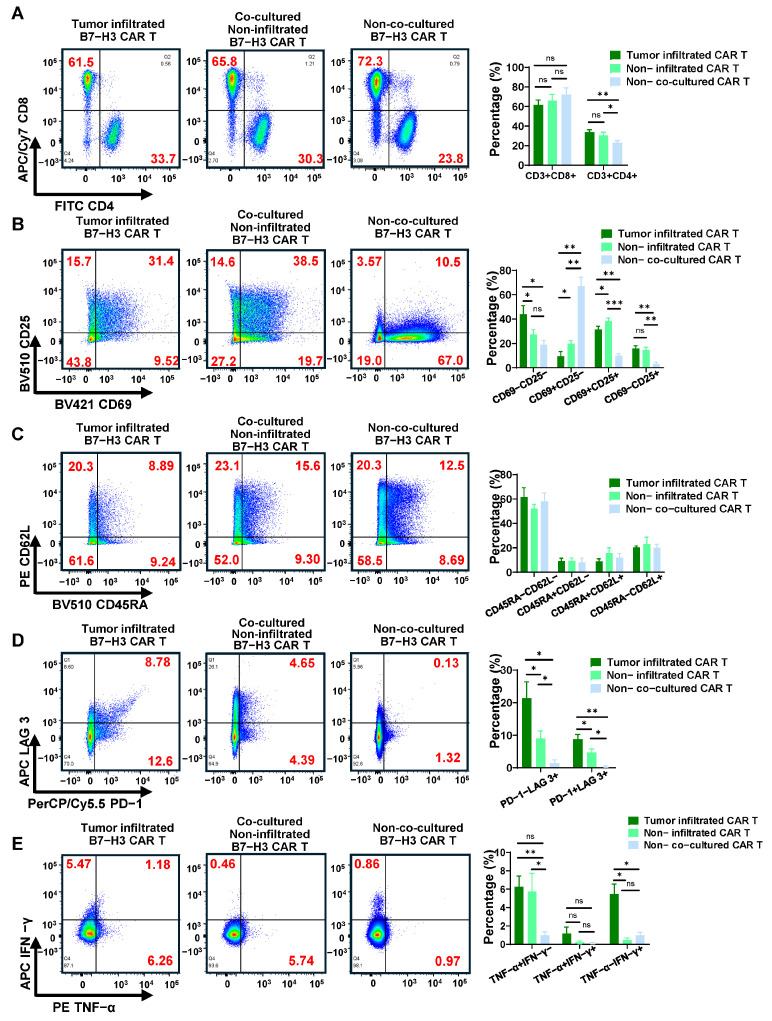
**Phenotypic and functional differences between tumor-infiltrated and non-infiltrated B7-H3 CAR T cells.** (**A**) CD4/CD8 distributions: CD3^+^CD8^+^ (effector T cells) and CD3^+^CD4^+^ (helper T cells), (**B**) activation status: CD69^−^CD25^−^ (resting T cells), CD69^+^CD25^−^ (initial activated T cells); CD69^+^CD25^+^ (fully activated T cells), andCD69^−^CD25^+^ (sustained activated T cells); (**C**) effector/effector memory subsets: CD45RA^−^CD62L^−^ (effector memory T cell), CD45RA^+^CD62L^−^ (effector T cell), CD45RA^+^CD62L^+^ (stem cell memory T cell), CD45RA^−^CD62L^+^ (central memory T cell); (**D**) PD-1 and LAG-3 expression: PD-1^−^LAG3^+^ (early exhaustion), PD-1^+^LAG3^+^ (late exhaustion); (**E**) IFN-γ and IFN-γ production. Two-way ANOVA was performed followed by Sidak’s multiple comparisons test. * indicates *p* < 0.05, ** indicates *p* < 0.01, *** indicates *p* < 0.001, ns = not significant.

First, flow cytometric analysis of CD45^+^CD3^+^ CAR T cells revealed significant subset redistribution following spheroid co-culture. Non-co-cultured CAR T cells exhibited a typical distribution with 23.8% CD4^+^ and 72.3% CD8^+^ cells. However, co-culture with tumor spheroids induced a notable shift toward increased CD4^+^ proportions, most pronounced within the tumor-infiltrating subset (33.7% CD4^+^) compared to non-infiltrating cells (Figure 5A). Correspondingly, CD8^+^ T cell proportions decreased to 65.8% in non-infiltrating and 61.5% in infiltrating populations.

This phenotypic shift directly correlates with our previous findings of upregulated MHC-II expression in 3D spheroids (Figure 2B), particularly the dramatic upregulation of MHC-II from 13.7% to 60.9% in DU 145 spheroids. The enhanced MHC-II presentation capacity likely provides stronger activation signals for CD4^+^ CAR T cells, promoting their preferential expansion and infiltration. Simultaneously, the observed MHC-I downregulation and APM defects may create a less favorable environment for CD8^+^ T cell activation, contributing to their relative reduction. However, because CAR signaling is designed to be MHC independent and TCR-mediated effects were not directly evaluated here, the specific contribution of altered MHC expression to CAR T cell function remains uncertain. Future studies incorporating untransduced T-cell controls, together with targeted analyses of TCR-dependent activation, will be important to more rigorously define how 3D culture–induced changes in MHC and APM expression influence CD4^+^/CD8^+^ composition and CAR T cell function.

Assessment of activation markers revealed that both non-infiltrating and spheroid-infiltrating CAR T cells demonstrated substantially elevated CD69^+^CD25^+^ double-positive cells (fully activated T cells, 38.5% and 31.4%, respectively) compared to non-co-cultured controls (10.5%) (Figure 5B), confirming robust antigen-driven activation. More importantly, spheroid-infiltrating CAR T cells exhibited the highest proportion of CD69^−^CD25^+^ cells (sustained activated T cells, 15.7% vs. 3.57% in controls), representing a sustained activation phenotype. However, this enhanced activation was counterbalanced by increased proportions of CD69^−^CD25^−^ cells (resting T cells) in spheroid-infiltrating CAR T cells (43.8%) compared to non-infiltrating (27.2%) and non-co-cultured (19.0%) populations (Figure 5B). This apparent paradox likely reflects the immunosuppressive nature of the spheroid microenvironment, where factors such as hypoxia, acidosis, and metabolic stress can promote T cell dysfunction despite ongoing antigen recognition. The elevated calreticulin expression observed in spheroids (Figure 2C) may contribute to this effect by inducing stress response pathways that ultimately limit sustained T cell activation.

Memory phenotype analysis showed alterations in T cell differentiation states following spheroid infiltration (Figure 5C). For instance, spheroid-infiltrating CAR T cells showed reduced proportions of CD45RA^+^CD62L^+^ T memory stem cells (TSCM), a critical subset for long-term therapeutic persistence due to their self-renewal capacity and multipotency. This reduction suggests that the tumor microenvironment drives premature differentiation away from highly desirable stem-like states.

Analysis of exhaustion markers revealed concerning patterns that highlight major challenges in solid tumor CAR T cell therapy. Spheroid-infiltrating CAR T cells exhibited the highest frequency of PD-1^+^ cells (21.4%) compared to free-floating (9.04%) and non-co-cultured (1.45%) populations (Figure 5D). More critically, the proportion of PD-1^+^LAG-3^+^ double-positive cells, representing severely exhausted T cells with compromised effector function, was dramatically elevated in tumor-infiltrating CAR T cells (8.78%) compared to non-infiltrating (4.65%) and control (0.13%) cells. This rapid exhaustion phenotype acquisition within the 3D environment suggests chronic antigen exposure, combined with inhibitory signals from the altered immune landscape we characterized, appears to drive CAR T cells toward dysfunction states that may limit therapeutic durability.

Intracellular cytokine analysis provided the most striking evidence for location-dependent CAR T cell functional states. Spheroid-infiltrating CAR T cells exhibited the highest functional activity, characterized by robust TNF-α (6.26%) and IFN-γ (5.47%) production, along with polyfunctional TNF-α^+^IFN-γ^+^ subsets (1.18%) (Figure 5E). This polyfunctional cytokine profile represents an effective anti-tumor T cell response, indicating that direct tumor contact and infiltration are essential for full CAR T cell activation. In contrast, non-infiltrating CAR T cells produced primarily TNF-α (5.74%) with minimal IFN-γ (0.46%), while non-co-cultured controls remained largely cytokine-negative. This spatial gradient of functional activity suggests that effective CAR T cell therapy requires not only target recognition but also successful infiltration into tumor tissues to achieve optimal Th1/CTL-like activation states.

These phenotypical and functional analyses demonstrate consistency with our previous microenvironmental characterizations. The observed CAR T cell phenotypic changes, including activation state alterations, and rapid exhaustion acquisition, directly reflect the complex immune landscape within 3D spheroids: upregulated MHC-II expression promoting CD4^+^ T cell responses, stress-induced calreticulin signaling affecting activation dynamics, and overall immunosuppressive environment driving exhaustion phenotypes. Future studies will incorporate longitudinal sampling to characterize time-dependent changes in CAR T-cell activation, exhaustion, and cytokine profiles in 3D spheroid models. The 3D spheroid model system proves importance for CAR T functional analyses by enabling isolation and detailed characterization of CAR T cells based on their microenvironmental location and exposure, making it essential for understanding and ultimately overcoming the unique challenges of solid tumor CAR T cell therapy.

## 4. Conclusions

This study establishes a physiologically relevant 3D tumor spheroid platform that successfully recapitulates key solid tumor microenvironmental features critical for evaluating CAR T cell therapeutic efficacy. Three-dimensional DU 145 and SUM 159 spheroids demonstrate enhanced B7-H3 target antigen expression. Immune profiling revealed that 3D culture induces significant alterations in tumor immune recognition machinery, including coordinated MHC-I and APM downregulation coupled with MHC-II upregulation and stress-associated calreticulin induction. These changes create a complex immunosuppressive landscape that closely mirrors primary solid tumor characteristics absent in conventional 2D culture systems. B7-H3 CAR T cells demonstrated robust antigen-specific cytotoxicity and progressive infiltration capability within this challenging microenvironment, achieving deep spheroid penetration and sustained killing activity over extended timeframes. Tumor-infiltrating CAR T cells rapidly acquire exhaustion phenotypes (elevated PD-1^+^LAG-3^+^ populations), undergo premature differentiation away from memory stem cell states, and experience altered CD4/CD8 subset dynamics reflecting the modified MHC expression landscape. This 3D tumor spheroid-based CAR T analysis provides critical insights into CAR T cell-tumor microenvironment interactions and offers a valuable preclinical tool for optimizing solid tumor immunotherapies, particularly for developing strategies to enhance infiltration while preventing exhaustion-mediated functional decline.

## Figures and Tables

**Table 1:** Marker panels.

Marker Panels	Markers
Panel 1	CD45-APC (cat# 304037, clone: HI30)
	CD3-PE/Cy7 (cat# 300420, clone: UCHT1)
	CD8-APC-Cy7 (cat# 344714, clone: SK1)
	CD4-FITC (cat# 357406, clone: RM4-5)
	CD25-BV510 (cat# 302640, clone: BC96)
	CD69-BV421(cat# 310930, clone: FN50)
Panel 2	CD45-APC
	CD3-PE/Cy7
	CD8-APC-Cy7
	CD4-FITC
	CD45RA-BV510 (cat# 304142, clone: HI100)
	CD62L-PE (cat# 304806, clone: MEL-14)
Panel 3	CD45-PE/Cy5 (car#368556, clone:2D1)
	CD3-PE/Cy7
	CD8-APC-Cy7
	CD4-FITC
	PD-1-PerCP/Cyanine5.5 (cat# 621614, clone: EH12.2H7)
	LAG-3-APC (cat# 369212, clone: 7H2C65)
Panel 4	CD45-PE/Cy5
	CD3-PE/Cy7
	CD8-APC-Cy7
	CD4-FITC
	TNF-α-PE (cat# 502909, clone: MAb11)
	IFN-γ-APC (cat# 502512, clone: 4S.B3)

## Data Availability

The data in the current study is available from the corresponding authors upon reasonable request.
